# The Role of Flavonoids in Inhibiting Th17 Responses in Inflammatory Arthritis

**DOI:** 10.1155/2018/9324357

**Published:** 2018-03-05

**Authors:** Dimitra Kelepouri, Athanasios Mavropoulos, Dimitrios P. Bogdanos, Lazaros I. Sakkas

**Affiliations:** ^1^Department of Rheumatology and Clinical Immunology, Faculty of Medicine, School of Health Sciences, University of Thessaly, 40500 Larissa, Greece; ^2^Division of Liver Transplantation and Mucosal Biology, King's College London School of Medicine, Denmark Hill Campus, London SE5 9RS, UK

## Abstract

Flavonoids have been considered powerful anti-inflammatory agents, and their exact immunomodulatory action as therapeutic agents in autoimmune diseases has started to emerge. Their role in the manipulation of immunoregulation is less understood. Several studies attempted to investigate the role of various flavonoids mainly in experimental models of autoimmune diseases, especially in the context of their potential effect on the increase of regulatory T cells (Tregs) and their ability to stimulate an overexpression of anti-inflammatory cytokines, in particular that of IL-10. The emergence of IL-17, a cytokine largely produced by Th17 cells, as a powerful proinflammatory stimulus which attenuates the induction of Tregs has prompted a series of studies investigating the role of flavonoids on Th17 cells in experimental models as well as human autoimmune diseases. This review thoroughly discusses accumulated data on the role of flavonoids on Th17 in rheumatoid arthritis and experimental autoimmune arthritis.

## 1. Introduction

Rheumatoid arthritis (RA) is the prototype of inflammatory chronic polyarthritis and is characterized by infiltration of T cells, B cells, macrophages, and fibroblasts in the synovial membrane, culminating in joint destruction and loss of function [1, 2]. The serological hallmark of the disease is the presence of high-titre rheumatoid factor (RF) and anticitrullinated peptide/antigen antibodies (ACPAs) [3–5].

## 2. The Role of Th17 Immune Response in RA Pathogenesis

The etiology of RA remains elusive; however, it is well recognized that CD4 T cells play a critical role in its pathogenesis as they heavily infiltrate the synovial membrane during RA synovitis [6]. This “T cell-centric theory” of RA pathogenesis has been challenged in the recent years as CD4 T cell depletion therapy failed to improve RA in clinical trials and the lack of T helper 1- (Th1-) related cytokines paradoxically exacerbated arthritis in some animal models (the “Th1 paradox”). As a result, a new proposition emerged where the key mediators of RA are the proinflammatory cytokines derived from macrophages and fibroblast-like synoviocytes, like TNF-*α*, IL-1, and IL-6 [7]. This resulted in the successful application of “anticytokine therapy,” such as the anti-TNF-*α* therapy or the anti-IL-6 therapy, which has revolutionized current RA treatment [8].

From 2005 onwards, the discovery of Th17 cells added significant insight into how T cells participate in the initiation and perpetuation of RA [9, 10]. This led to the proposal of a new “Th17 cell-centric theory” and revived the interest on CD4 T cells, which were found to produce IL-17 in the RA synovium. Elegant studies in animal models (see below) revealed that Th17 cells are a lineage of CD4 T cells distinct from classical Th1 or Th2 cells and play significant roles in autoimmune and inflammatory diseases [11]. The Th17 cells express the master transcription factor ROR*γ*t, are differentiated in vitro by TGF-*β* and IL-6, and can expand in the presence of IL-23, IL-1, and TNF-*α* [12]. Th17 differentiation may be cross-regulated by Th1, and, hence, the deficiency of Th1 cytokines led to the excessive differentiation of Th17 cells and paradoxically exacerbated RA in the animal models [13]. The advent of Th17 cells shed significant light in understanding the pathogenesis of RA.

Th17 cells are potent mediators of arthritis, which coordinate tissue inflammation, cartilage damage, and bone erosion. The arthritogenic potential of Th17 cells is mainly due to the pleiotropic effects of IL-17A (IL-17), which is produced by Th17 cells and acts on a variety of cells that constitute the synovial tissue [14, 15]. IL-17 synergistically enhances the production of TNF-*α*, IL-1, and IL-6 by macrophages and synovial fibroblasts. Additionally, IL-17 recruits neutrophils to the site of inflammation and promotes osteoclast differentiation, which leads to bone erosion and cartilage destruction. Two other cytokines produced by Th17 cells, IL-22 and IL-21, can alter the glycosylation of autoantibodies and grant them with inflammatory properties [16]. Th17 cells exhibit plasticity, that is, can shift to Th17/Th1 cells (producing both IL-17 and IFN-*γ*) and to Th1 cells (producing IFN-*γ*, so-called nonclassic Th1 cells) [17, 18]. These Th17-derived Th1 cells (transdifferentiated, nonclassic Th1 cells) appear to be more pathogenic than Th17 cells [19].

The activation of highly inflammatory Th17 cells is controlled under physiological conditions by regulatory T cells (Tregs) in order to prevent the development of autoimmune diseases [20, 21]. Tregs express the master transcription factor Foxp3 and upregulate the expression of CD25 and CTLA-4 on their surfaces in order to suppress the activation of effector CD4 T cells in a cell contact-dependent manner. There are two types of Tregs: naturally occurring Tregs (nTregs), which are derived from the thymus, and induced Tregs (iTregs), which are induced to differentiate from naïve T cells in the periphery and express the potent immunomodulatory cytokine IL-10 [22].

## 3. Animal Models of Experimental Arthritis and Th17 Cells

Most work on experimental models of inflammatory arthritis has been performed using type II collagen-induced arthritis (CIA) mice, SKG mice, and TNF-*α* transgenic mice [23–26]. These studies confirm the significant role of IL-17 in inflammatory arthritis.

### 3.1. Type II Collagen-Induced Arthritis Mice

Genetically susceptible strains of mice, such as C57BL/6 and DBA/1 mice, when injected with type II collagen in complete Freund's adjuvant, induced synovitis and erosion that histologically resembled RA [27, 28].

Sera of these mice contain antibodies against type II collagen, which can induce arthritis in other mice (CAIA, anti-type II collagen antibody-induced arthritis). IL-23-driven CD4 T cells, but not IL-12-driven Th1 cells, are the key mediators of CIA. The IL-23-driven CD4 T cells secreted IL-17 (Th17 cells) [29, 30]. Th17 cells contributed not only to joint inflammation but also to osteoclast differentiation and bone destruction.

### 3.2. SKG Mice

These mice are generated on a BALB/c genetic background and spontaneously develop autoimmune arthritis that resembles human RA [24, 31]. Similar to CIA, SKG arthritis is dependent on proinflammatory cytokines such as IL-6 and particularly IL-17 [32–35]. SKG CD4 T cells that were deficient in IL-17 failed to induce arthritis upon adoptive transfer into RAG2-deficient mice, while the induction of arthritis was accelerated by the transfer of IFN-*γ*-deficient CD4 T cells [35].

### 3.3. K/BxN Mice

These mice express both the T cell receptor (TCR) transgene KRN and the MHC class II molecule A(g7) (K/BxN mice) and develop severe inflammatory arthritis [15, 36–38].

### 3.4. TNF-*α* Transgenic Mice

DBA/1 mice expressing a human TNF-*α* transgene develop a severe form of erosive arthritis [39–41]. Although anti-IL-17 therapy had only minor effects on joint inflammation induced by TNF-*α*, it effectively reduced bone erosion in TNF-*α* transgenic mice [42, 43]. This result suggested that although arthritis in TNF-*α* transgenic mice can develop in a T cell-independent manner, IL-17 may also contribute to bone destruction, which is mediated by TNF-*α* [44].

### 3.5. gp130 F759/F759 Knock-In Mice

Glycoprotein 130 (gp130) mediates signal transduction by IL-6 family cytokines such as IL-6, IL-27, and IL-35, through the signal transducer and activator of transcription 3 (STAT3) and/or Src homology region 2 domain-containing phosphatase 2 (SHP2) signaling. In this mouse model [45], Th17 cells are expanded as IL-6/gp130 signaling promotes Th17 differentiation [46].

### 3.6. IL-1 Receptor-Antagonist Knockout Mice

IL-1RA-deficient mice on a BALB/c background spontaneously developed chronic inflammatory polyarthritis [47, 48]. Overexpression of IL-1 leads to expansion of Th17 cells. As a result, the expression of IL-17 or IL-23 is greatly enhanced in IL-1RA KO mice, whereas arthritis development is inhibited during IL-17 deficiency or IL-23 blockade [49–51].

Studies of the impact of novel treatment/regimen, including diet complements, are usually studied in animal models, such as those mentioned above, the great majority of those conducted using the CIA model. Elegant reviews have been published discussing the current animal models of RA which summarize the pros and cons of each model as well as the role played by Th17 in the induction and perpetuation of inflammatory arthritis [11] (Table 1).

## 4. Flavonoids: An Overview

By the term *flavonoids*, we refer to a broad class of compounds that are defined by color (pigment). Literally, the term originates from the Latin word *flavus*, which means yellow. Flavonoids are a group of secondary plant metabolites present in the diet with cholesterol-lowering, antioxidant, and other health-beneficial biological activities [52–54]. They are also commonly found in seeds, nuts, grains, and spices and in some beverages, such as wine, tea, and beer [55].

**Figure 1:**
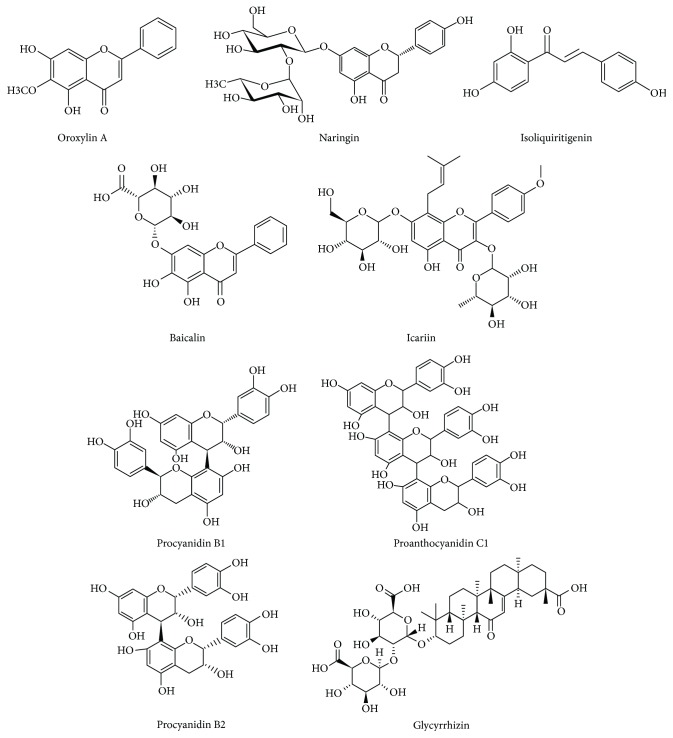
Chemical structures of principal flavonoids discussed in relation to their role on Th17 in rheumatoid arthritis and its experimental models.

Chemically, flavonoids are polyphenols conjugated to sugars (as a glycosylated form) although some can exist as free aglycones. The basic flavonoid structure is the flavan nucleus consisting of a 15-carbon skeleton arranged in two phenyl rings bound by a three-carbon bridge commonly encircled by oxygen, then forming three rings (Figure 1) [56]. The main classes of flavonoids are flavonols, flavones, flavanones, flavanols, isoflavones, and anthocyanidins [57].

Traditional Chinese medicine used over the last 3000 years to treat, manage, or prevent human diseases has largely been based on the beneficial role of natural bioactive compounds from herbs containing flavonoids [58]. A typical example is that of licorice, also called Gancao in China, derived from the dried roots and rhizomes of the *Glycyrrhiza* species, which is used to treat diabetes, tuberculosis, and other inflammatory disorders not only in China but also in Korea, Japan, and India [59]. Licorice is probably the most frequently used herbal medicine worldwide, as Gancao appears in over 50% of traditional Chinese medicine prescriptions and confectionery products [60]. The large number of natural compounds containing flavonoids and the wide range of cell signaling pathways involved make it extremely difficult to simplify the anti-inflammatory role of flavonoids in various inflammatory diseases. Such studies are elegantly reviewed elsewhere [61].

The anti-inflammatory activity of flavonoids is exerted through various mechanisms, mostly shared by most flavonoid compounds, and these largely include the direct or indirect inhibition of proinflammatory cytokines through the immunomodulation of key inflammatory signaling cascades, the diminished recruitment of proinflammatory cell subsets, their increased antioxidant properties, and their beneficial effect on immunoregulatory functions.

Recent evidence has suggested that flavonoids can be potential modifiers of innate and adaptive immunity [62–64]. Immune system impairment accounts for the increased risk of infections, inflammatory chronic disease, and autoimmunity. Flavonoids and polyphenols can target multiple inflammatory components and reinforce anti-inflammatory mechanisms with antioxidant potential [52, 54, 65]. Certain flavonoids, namely, quercetin, apigenin, and luteolin, reduce cytokine expression and secretion [66]. In this regard, flavonoids may have therapeutic potential in the treatment of inflammation-related diseases as cytokine modulators. TLR suppression, PI3K/Akt inhibition, IKK/MAPK inhibition, mTORC1 inhibition, NF*κ*Β, and JAK/STAT inhibition have been attributed as targets of flavonoid-mediated suppression of inflammation [67–69].

Although the immunomodulatory potential of flavonoids has been investigated to some extent, an established effect of these compounds in clinical trials has been controversial [70]. This is due to the diversity in their subclasses, as well as the unresolved problems related to the purity and the selected doses of these compounds. Nevertheless, current research in animal models and preclinical studies are promising and warrant further investigation of these compounds. However, very little is known about the impact of flavonoids on Th17-related immune modulation and their potential effect on autoimmune rheumatic disorders, such as RA. Some principal nutraceuticals that can modify the Th17 immune response and have shown some effects in animal models of RA are illustrated in Table 2.

### 4.1. Oroxylin A

Oroxylin A is one of the many flavonoid glycosides extracted from the plant *Scutellaria baicalensis radix* and the *Oroxylum indicum* tree bark but the only O-methylated flavone [71, 72]. Methylation provides flavonoids with increased metabolic stability and delays their hepatic metabolism. It also improves their intestinal absorption, ensuring better bioavailability.

In mice with induced arthritis, oroxylin A inhibited the production of inflammatory cytokines IL-1*β*, IL-6, TNF-*α*, and IL-17. TNF-*α*-induced p38 MAPK, ERK1/2, and NF-*κ*B signaling pathways were also suppressed [73, 74]. Oroxylin A also increased the production of Tregs and reduced Th17 cells in the lymph nodes draining arthritis joints in mice with CIA [73] (Table 2). Oroxylin A suppressed the secretion of IL-1*β* and IL-6 from TNF-*α*-stimulated fibroblast-like synovial cells from RA patients. In TNF-*α*-stimulated RA fibroblast-like synovial cells, it also suppressed p38 MAPK and ERK-1/2 and prevented the nuclear translocation of NF-*κ*B p65 [73].

Oroxylin A inhibits NO, cytokines, chemokines, and growth factors in induced macrophages via the calcium-STAT pathway and exerts an anti-inflammatory activity on lipopolysaccharide-induced mouse macrophages via Nrf2/ARE activation [75]. Inhibition of lipopolysaccharide-induced iNOS and COX-2 gene expression was also mediated via suppression of NF-*κ*B [76].

### 4.2. Baicalin

Baicalin is a flavonoid compound isolated from the dry root of *Scutellaria baicalensis* Georgi (Huang-Qin), a medicinal plant. Baicalin is a flavone glycoside, glycosylated in the 7-position, glucuronide of baicalein. Baicalin inhibits Th17 cell differentiation *in vitro* and upregulates Foxp3^+^ Tregs [77]. It also inhibits Th17 cell differentiation in vivo in lupus-prone MRL/lpr mice [77]. Baicalin reduced splenic Th17 cells and ameliorated murine adjuvant-induced arthritis without affecting Tregs. Furthermore, it significantly blocked IL-17-stimulated synoviocyte gene expression of ICAM-1, VCAM1, IL-6, and TNF-*α* [78]. Baicalin blocked CIA in rats and inhibited the secretion of IL-1*β* and TNF-*α* in rat synovium [79]. Baicalin also prevented Th1 and Th17 cell differentiation via STAT/NF-*κ*B signaling pathways and ameliorated clinical disease severity in experimental autoimmune encephalomyelitis (EAE) [80]. Furthermore, it suppressed Th17 development by upregulating the suppressor of cytokine signaling 3 (SOCS3) [80].

UP446, a composition consisting primarily of baicalin from *Scutellaria baicalensis* Georgi and catechin from the heartwoods of *Acacia catechu*, has been previously shown to reduce the production of eicosanoids and leukotrienes through dual inhibition of COX and lipo-oxygenase (LOX) enzymes and to decrease mRNA and protein levels of IL-1*β*, IL-6, and TNF-*α*, suggesting a potential benefit of UP446 in alleviating symptoms of RA and support further assessment of this botanical composition in patients with RA [81].

A combination of flavonoids from the *Scutellaria* root has been found to inhibit PGE2 production more potently than individual flavonoids do. The synergistic effect of the flavonoid mixture of baicalin and oroxylin A reflected a broad action in inhibiting multiple steps in the NF-*κ*B signaling pathway [71].

### 4.3. Icariin

Icariin is a prenylated flavonol glycoside, a subclass of flavonoids. Prenylated flavonoids occur when the flavonoid ring is substituted by prenyl groups. This provides them a stronger adherence to cell membranes and enhances lipophilicity. From the various flavonoid glycosides of the genus *Epimedium*, icariin is the most metabolically active constituent and is obtained from the aerial part of the plant [82]. The difference between flavonols and flavones (oroxylin, baicalin) is that the former possess a hydroxyl group in the 3 position and can be regarded as 3-hydroxyflavones.

Icariin can suppress cartilage and bone degradation in mice with CIA [83]. It appears that icariin can exert its effects through a profound reduction of Th17 cells and IL-17 production, through inhibition of the STAT3 pathway [84]. Icariin inhibited the progression of the disease in a dose-dependent manner.

The effect of icariin on CIA is not unexpected. It has been reported that icariin has antiosteoporotic, anti-inflammatory, and antidepressant-like activities [85]. Its role on Th17/Treg balance was also reported in airway inflammation of an ovalbumin- (OVA-) induced murine asthma model [86]. Icariin decreased the inflammatory cells infiltrating the peribronchial tissues and mucus hyperproduction. This was associated with reduction in CD4^+^ROR*γ*t^+^ T cells and increase in CD4^+^Foxp3^+^ T cells in bronchial-alveolar lavage fluid (BALF). Furthermore, icariin caused a significant reduction in IL-6, IL-17, and TGF-*β* levels in BALF. Also, icariin inhibited Th1 and Th17 cell differentiation and ameliorated EAE [87]. Finally, in mice with dextran sulfate sodium-induced colitis, icariin attenuated disease and inhibited the activation of STAT1 and STAT3, transcription factors of Th1 and Th17, respectively [88].

### 4.4. Procyanidins B1, B2, and C1 from Apples

Apples contain high concentrations of phytochemicals, phenolic compounds, and condensed tannins, including procyanidins B1, B2, and C1 [89]. Procyanidins are members of the proanthocyanidin (or condensed tannins) class of flavonoids. In fact, the most common subclass of proanthocyanidins are procyanidins, which are made of elementary flavan-3-ol (epi)catechin units.

Procyanidin B2 (PCB2) inhibits the production of proinflammatory cytokines in macrophages. PCB2 gallates inhibited the activation and proliferation of T cells after stimulation with anti-CD3 mAb and reduced the production of interferon- (IFN-) *γ*, IL-12p40, and IL-17 in splenocytes, but not IL-10 production [90]. DBA1/J mice with CIA fed with apple-condensed tannins exhibited a significant delay in the appearance of arthritic symptoms. Apple-condensed tannins reduced the production of the proinflammatory IL-17 and IFN-*γ* cytokines [91].

### 4.5. Grape Seed Proanthocyanidins

Proanthocyanidins are polyphenolic compounds that can be found in the plant physiology of several plant species, mainly concentrated in tree barks and outer skins of seeds. Proanthocyanidins, commonly referred to as condensed tannins, are a class of flavanols, which belong to a larger group of polyphenolic compounds. Grape seed contains many polyphenolic compounds that have potential health-promoting benefits and is one of the richest sources of proanthocyanidins [92].

Grape seed proanthocyanidin extract (GSPE) treatment, in a dose-dependent manner, significantly reduced the severity of CIA and reduced the numbers of IL-17- and TNF-*α*-producing cells in the arthritic tissue and the spontaneous production of IL-17 and TNF-*α* by splenocytes. Furthermore, GSPE suppressed osteoclastogenesis *in vitro* in a dose-dependent manner [93]. GSPE from *Vitis vinifera* has potent antiarthritic effects on CIA by modifying the T cell homeostasis. Treg cells, which are important inhibitors of inflammation and mediators of self-tolerance, are deficient in RA. Park et al. showed that grape seed proanthocyanidins induce the development of Foxp3^+^ Tregs [94]. GSPE-treated mice had significantly increased CD4^+^CD25^+^Foxp3^+^ Tregs in vivo. The concomitant suppression of IL-17 production and the enhancement of Foxp3 expression by the GSPE in T cells of joints and splenocytes was associated with alleviation of established CIA. Furthermore, GSPE induced Foxp3^+^ Tregs and suppressed IL-17-, IL-21-, and IL-22-producing T cells in human T cell culture, and this was associated with the abrogation of STAT3 [94]. In another study, Ahmad et al. demonstrated that the administration of GSPE in mice with adjuvant-induced arthritis alleviated arthritis, and this was associated with an increase in Foxp3^+^ Tregs and decrease in Th17 and Th1 cells in peripheral blood and a decrease in IL-17A, IFN-*γ*, and TNF-*α* in the arthritic tissue [95].

In other models, proanthocyanidins from the bark of *Metasequoia glyptostroboides* (MGEB) ameliorated allergic contact dermatitis through direct inhibition of T cell activation and Th1/Th17 responses. More specifically, the anti-inflammatory activity of MGEB was evaluated using 2,4-dinitrofluorobenzene- (DNFB-) induced allergic contact dermatitis (ACD) in mice [96]. MGEB inhibited Con A-induced proliferation and the expression of cell surface CD69 and CD25 in T cells in vitro. MGEB also significantly decreased the production of Th1/Th17 cytokines (IL-2, IFN-*γ*, and IL-17) in activated T cells.

### 4.6. Licorice

Licorice is the root of *Glycyrrhiza glabra*, a medicinal plant famous for its sweet flavor. The licorice plant is perennial, native to southern Europe and parts of Asia, such as India. It is known as a potent medicine, effective against peptic ulcer, constipation, cough, and viral infection. The various pharmacological properties of licorice are attributed to triterpene saponins, such as glycyrrhizin, and flavonoids, such as liquiritin, isoliquiritin, and their aglycones. The plant's roots contain significant amounts of phenolic and flavonoid compounds.

Guo et al. showed that two flavonoids isolated from licorice, isoliquiritigenin and naringenin, have the capacity to increase the number of Tregs [97]. They can also promote Treg cell differentiation and enhance Treg cell function. Naringenin can promote Treg cell differentiation, as an aryl hydrocarbon agonist expressed in both Th17 and Treg cells, whereas isoliquiritigenin cannot. Licorice Gly1 promoted Treg differentiation in vitro [97]. In addition, licorice decreased the production of IL-2, an inflammatory cytokine produced by Th1 cells that promotes T cell proliferation and survival.

The ability of licorice-related flavonoids to exert anti-inflammatory responses through the inhibition of Th17 cells is well established in other models of autoimmune diseases. Glycyrrhizin, a component of Chinese medicine licorice root, has the ability to inhibit the functions of high-mobility group box 1 (HMGB1). Glycyrrhizin treatment of TNBS-induced murine colitis model decreased the production of proinflammatory mediators HMGB1, IFN-*γ*, IL-6, TNF-*α*, and IL-17. Furthermore, glycyrrhizin regulated the responses of dendritic cells (DCs) and macrophages and suppressed the proliferation of Th17 cells [98].

Naringenin is a flavanone glycoside found in grapes and citrus fruits (*Citrus paradisi*). The bitter flavor of grapefruit is attributed to this particular flavanone. Two rhamnose units are attached to its aglycon portion, naringenin, at the 7-carbon position. Both naringenin and naringin are strong antioxidants, with naringin being more potent. Both flavonoids block several inflammatory pathways, inhibiting inflammation and reducing oxidative stress [99]. Naringin is moderately soluble in water. Of interest, gut microflora breaks down naringin to its aglycon naringenin, which is then absorbed from the gut. In mice, naringenin reduced inflammatory pain by blocking the NF-*κ*B pathway. It also interferes with enzymatic activity in the intestines and, thus, with the breakdown of certain drugs. Drugs affected by naringin are calcium channel blockers, estrogen, sedatives, medications for high blood pressure, and cholesterol-lowering drugs. No data currently exist on the role of this compound on experimental or human arthritis in relation to Th17/Treg imbalance.

Isoliquiritigenin (ISL) is a flavonoid with a chalcone structure. It shows various biologic properties, including anti-inflammatory and antioxidative actions, as well as vasorelaxant and estrogenic effects. Of relevance, chalcones are considered to be important intermediates in flavonoids' synthesis. Their biological activities include those that are antiallergic, antiangiogenesis, and antitumor growth. At the cellular level, ISL inhibits various steps of angiogenesis, including VEGF-induced endothelial cell proliferation, tube formation, migration, and aortic ring sprout formation. ISL suppresses adipose tissue inflammation by affecting the paracrine loop containing saturated fatty acids and TNF-*α* in cocultures on adipocytes and macrophages [100] through inhibition of NF-*κ*B activation.

Whether ISL exerts any anti-inflammatory effect on RA or experimental arthritis remains unclear. Licochalcone A., derived from *Glycyrrhiza inflata*, reduced the clinical severity of EAE mice and inhibited IFN-*γ*, IL-17, and TNF-*α* production in peritoneal cells [101].

*Glycine max*, commonly known as soybean in North America or soya bean, is a species of legume native in Asia. Soybean is a valuable and popular crop globally and is used to produce a variety of products such as soy paste, soybean sprouts, soy curd, soy milk, tofu, and oil.

Isoflavones and anthocyanins, both of which are beneficial for human health, are found in soybean. Several major anthocyanins (cyanidin-3-glucoside, delphinidin 3-glucoside, and petunidin 3-glucoside) have been isolated from the seed coat of black soybeans. The effect of soya bean in RA via Th17 inhibition has not been thoroughly studied so far.

### 4.7. Anthocyanins

Anthocyanins are water-soluble members of the flavonoid group, which, depending on their pH, may appear red, purple, or blue. Food plants rich in anthocyanins include the blueberry, raspberry, black rice, and black soybean [102]. Anthocyanin is a representative antioxidant of the flavonoid family found in plants [57].

Black soybean seed coats are an excellent source of anthocyanin. Anthocyanins are synthesized via the phenylpropanoid pathway and can be found in all parts of the plant, including leaves, stems, roots, flowers, and fruits. Anthocyanins are derived from anthocyanidins by adding sugars. Anthocyanins are very unstable compared to other flavonoids, particularly at neutral or alkaline pH. They are detected mainly as unmetabolized glycosides in plasma and in urine, rather than as aglycones.

Anthocyanins can interfere with and inhibit the process of carcinogenesis; thus, they are often described as natural antioxidants. The chemical structure of flavonoids is also important in determining their bioactive properties [103].

Min et al. described the potent antiarthritic activity of anthocyanins from black soybeans in vivo [104]. Anthocyanins extracted from black soybeans (AEBS) exerted therapeutic effects in a RA mouse model both in vivo and *in vitro* and in humans *in vitro*. AEBS decreased Th17 cells both *in vitro* and in vivo and inhibited the expression of proinflammatory cytokines (TNF-*α*, IL-6, IL-17, IL-21) in mice with CIA by blocking the NF-*κ*B pathway. Finally, osteoclastogenesis was suppressed by AEBS in both DBA/1J mice and human cells in vitro.

## 5. Conclusion

In autoimmune arthritis, inflammatory Th17 cells producing IL-17 are inversely associated with anti-inflammatory regulatory T cells (Tregs). Flavonoids encompass various compounds present in traditional medicines, long used as therapeutic agents in autoimmune inflammatory diseases. The anti-inflammatory properties of flavonoids are increasingly elucidated *in vitro* and in animal models of arthritis, as flavonoids have been shown to inhibit cyclooxygenase, to reduce the production of inflammatory cytokines, to suppress p38 MAPK, to inhibit Th17 cells, and to increase Tregs.

## Figures and Tables

**Table 1:** Main features of animal models of rheumatoid arthritis regarding proinflammatory cytokines, including IL-17.

Animal models	IL-17	IL-23	IFN-*γ*	TNF-*α*	IL-6	References
CIA mice	+	+	++	+++	++	[27, 29, 30, 105]
SKG mice	++	++	+	+	+	[32–35]
K/BxN mice	++	+/−	++	+	+/−	[15, 37, 38]
TNF-*α* transgenic mice	+	+	+	+++	+	[39, 40, 43, 44]
gp130 F759/F759 knock-in mice	++	+	+	+	++	[45, 46]
IL-1 RA knockout mice	++	++	+	+	+	[47, 49–51]

^+^Low expression, ^++^moderate expression, and ^+++^high expression.

**Table 2:** Main features and findings of studies investigating the role of flavonoids in Th17 cells in animal models of rheumatoid arthritis.

Flavonoid name	Year of study	Experimental model	Administration and dosage	Biological finding	Clinical finding	References
Oroxylin A	2016	In male mice DBA/1 with type II collagen-induced arthritis	10 mg/kg oroxylin A for 10 days intraperitoneal	Increase of Tregs and reduction of Th17	Significant decline in arthritis and histological damage	[73]
Baicalin	2013	In male mice C57BL/6 (B6) with adjuvant-induced arthritis	100 mg/kg baicalin intraperitoneal for 7 days	Inhibition of splenic Th17 cells expansion in vivo. Inhibition of IL-17 inflammation in synoviocytes	Alleviation of inflammatory joint injury in mice with adjuvant-induced arthritis	[78]
Icariin	2014	In male mice C57BL/6 (B6) with type II collagen-induced arthritis	Oral dose of 25 mg/kg with icariin for 20 days	Decrease of Th17 cells and repression of IL-17 production	Suppression of inflammatory arthritis dependent on IL-17 production	[84]
Condensed tannins from apples	2015	In mice DBA1/J with type II collagen-induced arthritis	5–7 mL 1% w/v oral administration of ACT per day for 2 weeks	Reduction of IL-17 expression. Downregulation of Th17 development. Increase of Treg production	Delay in rheumatoid arthritis development in mice	[91]
Anthocyanins from black soybean seed	2015	In mice DBA1/J with type II collagen-induced arthritis	60 mg/kg oral administration of AEBS dissolved in saline for 7 weeks	Reduction of IL-17-expressing T cells. Inhibition of Th17 cell differentiation and Th17 cell differentiation-associated genes	Antiarthritic effects: decrease in incidence of arthritis, histological inflammation, cartilage scores, and oxidative stress	[104]
Grape seed proanthocyanidins	2011	In male mice DBA/1J with type II collagen-induced arthritis	300 mg/kg GSPE 3 times a week for 2 weeks	Repression of IL-17 in Th cells. Inhibition of Th17 cells. Induction of Foxp3 Treg cells. Inhibition of Th17-associated gene expression in human Th17 cells	Inhibition of the activity of autoimmune arthritis. Attenuation of the symptoms of collagen-induced arthritis	[94]
Grape seed proanthocyanidins	2013	In BALB/c female adult mice	25, 50, 100 mg/kg oral administration of GSPE once a day for 2 weeks	Increase of Foxp3^+^ Treg cells and reduction of Th17 cells. Upregulation of Th2 cells. Induction of Th17/Treg rebalance	Antiarthritic activity. Protection of GSPE against arthritis. Significant reduction in paw edema in mice	[95]

## Abbreviations

ACPA: Anticitrullinated peptide/antigen antibodies
CIA: Collagen-induced arthritis
GSPE: Grape seed proanthocyanidin extract
iTregs: Induced regulatory T cells
nTregs: Naturally occurring Tregs
RA: Rheumatoid arthritis
RF: Rheumatoid factor
Th: T helper cells.
